# Sleep-Disordered Breathing, Advanced Age, and Diabetes Mellitus Are Associated with De Novo Atrial Fibrillation after Cardiac Surgery

**DOI:** 10.3390/biomedicines12051035

**Published:** 2024-05-08

**Authors:** Maria Tafelmeier, Sabrina Kuettner, Christian Hauck, Bernhard Floerchinger, Daniele Camboni, Marcus Creutzenberg, Florian Zeman, Christof Schmid, Lars Siegfried Maier, Stefan Wagner, Michael Arzt

**Affiliations:** 1 Department of Internal Medicine II (Cardiology, Pneumology, and Intensive Care), University Medical Center Regensburg, 93053 Regensburg, Germany; sabrina.kuettner@stud.uni-regensburg.de (S.K.); christian.hauck@klinik.uni-regensburg.de (C.H.); lars.maier@klinik.uni-regensburg.de (L.S.M.); stefan.wagner@klinik.uni-regensburg.de (S.W.); michael.arzt@klinik.uni-regensburg.de (M.A.); 2 Department of Cardiothoracic Surgery, University Medical Center Regensburg, 93053 Regensburg, Germany; bernhard.floerchinger@klinik.uni-regensburg.de (B.F.); daniele.camboni@klinik.uni-regensburg.de (D.C.); christof.schmid@klinik.uni-regensburg.de (C.S.); 3 Department of Anesthesiology, University Medical Center Regensburg, 93053 Regensburg, Germany; marcus.creutzenberg@klinik.uni-regensburg.de; 4 Center for Clinical Studies, University Medical Center Regensburg, 93053 Regensburg, Germany; florian.zeman@klinik.uni-regensburg.de

**Keywords:** coronary artery bypass grafting (CABG), coronary artery disease, sleep apnea, postoperative complications

## Abstract

**Background:** Postoperative de novo atrial fibrillation (POAF) is one of the most frequently encountered complications following cardiac surgery. Despite the identification of several risk factors, the link between sleep-disordered breathing (SDB) and POAF has barely been examined. The objective of this prospective observational study was to determine whether severe SDB is associated with POAF in patients after elective coronary artery bypass grafting (CABG) surgery. **Study design and methods:** The incidence and preoperative predictors of in-hospital POAF were assessed in 272 patients undergoing CABG surgery at the University Medical Center Regensburg (Germany). In-hospital POAF was detected by continuous telemetry-ECG monitoring and 12-lead resting ECGs within the first seven postoperative days. POAF that occurred after hospital discharge within 60 days post CABG surgery was classified as post-hospital POAF and was ascertained by standardized phone interviews together with the patients’ medical files, including routinely performed Holter-ECG monitoring at 60 days post CABG surgery. The night before surgery, portable SDB monitoring was used to assess the presence and type of severe SDB, defined by an apnea–hypopnea index ≥ 30/h. **Results:** The incidence of in-hospital POAF was significantly higher in patients with severe SDB compared to those without severe SDB (30% vs. 15%, *p* = 0.009). Patients with severe SDB suffered significantly more often from POAF at 60 days post CABG surgery compared to patients without severe SDB (14% vs. 5%, *p* = 0.042). Multivariable logistic regression analysis showed that severe SDB (odds ratio, OR [95% confidence interval, CI]: 2.23 [1.08; 4.61], *p* = 0.030), age ≥ 65 years (2.17 [1.04; 4.53], *p* = 0.038), and diabetes mellitus (2.27 [1.15; 4.48], *p* = 0.018) were significantly associated with in-hospital POAF. After additional adjustment for heart failure, the association between sleep apnea and postoperative atrial fibrillation was attenuated (1.99 [0.92; 4.31], *p* = 0.081). **Conclusions:** Amongst established risk factors, severe SDB was significantly associated with in-hospital POAF in patients undergoing CABG surgery. Whether SDB contributes to POAF independently of heart failure and whether risk for POAF may be alleviated by proper treatment of SDB merits further investigation.

## 1. Background

Postoperative de novo atrial fibrillation (POAF) is highly prevalent and affects approximately 25% of patients undergoing isolated coronary artery bypass grafting (CABG) surgery [1,2,3]. In-hospital POAF usually manifests on postoperative day 2, with 70% of cases occurring within the first seven postoperative days [4]. POAF usually has self-limiting characteristics with an average duration of 11–12 h [4]. However, almost half of the patients experience a second episode, and nearly a quarter of the patients suffer from recurrent episodes of POAF [5].

Clinical manifestations of POAF in patients undergoing CABG surgery predominantly include hypotension, tachyarrhythmia, palpitations, and shortness of breath. These symptoms may rapidly progress into hemodynamic instability, reduced cardiac output, and heart failure [4]. Thus, POAF has been associated with increased morbidity and mortality among patients undergoing CABG surgery [6,7], significantly affects hospital resources, and incurs considerable costs [6].

Numerous studies have shown that POAF is associated with advanced age, male gender, obesity, chronic obstructive pulmonary disease, heart failure, and left atrial enlargement [4,6]. Although various risk factors for POAF have been identified, the prevention and optimal management of POAF remain a major challenge within the perioperative care of CABG patients. 

Sleep-disordered breathing (SDB) is a common disorder that is estimated to affect approximately 17% of men and 9% of women aged between 50 and 70 years [8,9]. Due to shared risk factors, the prevalence of SDB is particularly high in patients with coronary artery disease [10,11]. SDB contributes to the occurrence of cardiac arrhythmias [12], and epidemiologic studies have recognized a strong association between both obstructive (OSA) and central sleep apnoea (CSA) and atrial fibrillation (AF), with SDB increasing the risk for AF by 2–4 times [13,14]. In contrast to CSA, OSA has been linked to an increased recurrence of AF after pulmonary vein isolation that may be alleviated by CPAP treatment [15].

To date, several prospective observational studies and meta-analyses have addressed OSA as an important predictor of POAF in patients undergoing CABG surgery [16,17,18,19,20]. However, previous studies are limited by small study populations and the use of diagnostic tools (e.g., questionnaires) that do not allow the assessment of the type and severity of SDB [19,20]. Remarkably, CSA has never been taken into account, although the prevalence of CSA is high in this patient population and should not be neglected in POAF analyses [21,22]. Therefore, the objectives of the present study were to determine whether severe SDB is associated with in-hospital POAF in patients undergoing elective CABG surgery. 

## 2. Methods

### 2.1. Study Design and Patients

The present sub-analysis is part of the ongoing prospective observational clinical study ‘Impact of Sleep-disordered breathing on Atrial Fibrillation and Perioperative complications in Patients undergoing Coronary Artery Bypass grafting Surgery’ (CONSIDER AF, NCT02877745) that evaluates the impact of SDB on the rate of Major Adverse Cardiac and Cerebrovascular Events in patients undergoing elective CABG surgery at the Department of Cardiothoracic Surgery of the University Medical Center Regensburg, Germany [23,24]. This study was approved by the Ethics Committee of the University of Regensburg (no. 15-101-0238). 

Between May 2016 and May 2019, elective patients aged between 18 and 85 years were tested for eligibility. Informed consent was obtained from all eligible patients who were willing to participate in the study. As outlined beforehand [24], exclusion criteria were severe obstructive pulmonary disease, oxygen therapy, nocturnal positive airway pressure support or mechanical ventilation, and the need for catecholamines or circulatory assist devices [24]. Additionally, patients with a history of AF were omitted from this sub-analysis to ensure that only POAF was detected.

### 2.2. SDB-Monitoring and Postoperative Treatment

The night before CABG, portable SDB monitoring was conducted using the Alice NightOne device (Philips Respironics, Murrysville, PA, USA), equipped with three sensors measuring nasal flow, pulse oximetry, and thoracic breathing effort [23,24]. Trained medical personnel scored the data collected by the Alice NightOne devices using the Sleepware G3 sleep diagnostic software (Philips Respironics, Murrysville, PA, USA). For additional information, please consult the online data supplement. 

### 2.3. Assessment of Postoperative De Novo Atrial Fibrillation

AF was defined according to the guidelines of the European Society of Cardiologists; that is, any arrhythmia that presents the electrocardiographic characteristics of AF within a standard 12-lead ECG or a single-lead ECG tracing of ≥30 s [25]. In-hospital POAF was diagnosed through continuous telemetry-ECG monitoring and by 12-lead resting ECGs performed within the routine postoperative care during the first seven postoperative days. AF that occurred after hospital discharge within 60 days post CABG surgery was classified as post-hospital POAF and was ascertained by standardized phone interviews together with the patients’ medical files, including routinely performed Holter-ECG monitoring. Predisposing risk factors for in-hospital POAF, such as demographics, common comorbidities, and laboratory and perioperative data, were assessed by means of the patients’ clinical records. 

### 2.4. Data Management and Statistical Analysis

The data management and statistical analysis for this sub-analysis adhered to the data handling plan outlined in the published study protocol of the CONSIDER-AF study [24]. SPSS 26.0 (IBM, New York, NY, USA) was used for statistical analyses. Data were summarized as mean ± standard deviation for normally distributed data and as median (25th; 75th percentile) for non-normally distributed data, while categorical variables were described as absolute and relative frequencies. Group comparisons utilized various statistical tests: Student’s *t*-test or ANOVA for normally distributed continuous variables, Mann–Whitney U test or Kruskal–Wallis test for non-normally distributed continuous variables, and Pearson’s chi-square test for categorical variables. Univariable logistic regression analyses assessed predisposing risk factors as independent variables and in-hospital de novo AF as the dependent variable. Additionally, three multivariable logistic regression models were constructed, progressively integrating demographic parameters (Model I [demographic model]), established comorbidities (Model II [comorbidity model]), and a variable representing heart failure (Model III [heart failure model]). Effect estimates were expressed as odds ratios (OR) with corresponding 95% confidence intervals (CI). A significance threshold of *p* ≤ 0.05 was applied for all analyses.

## 3. Results

**Figure 1:**
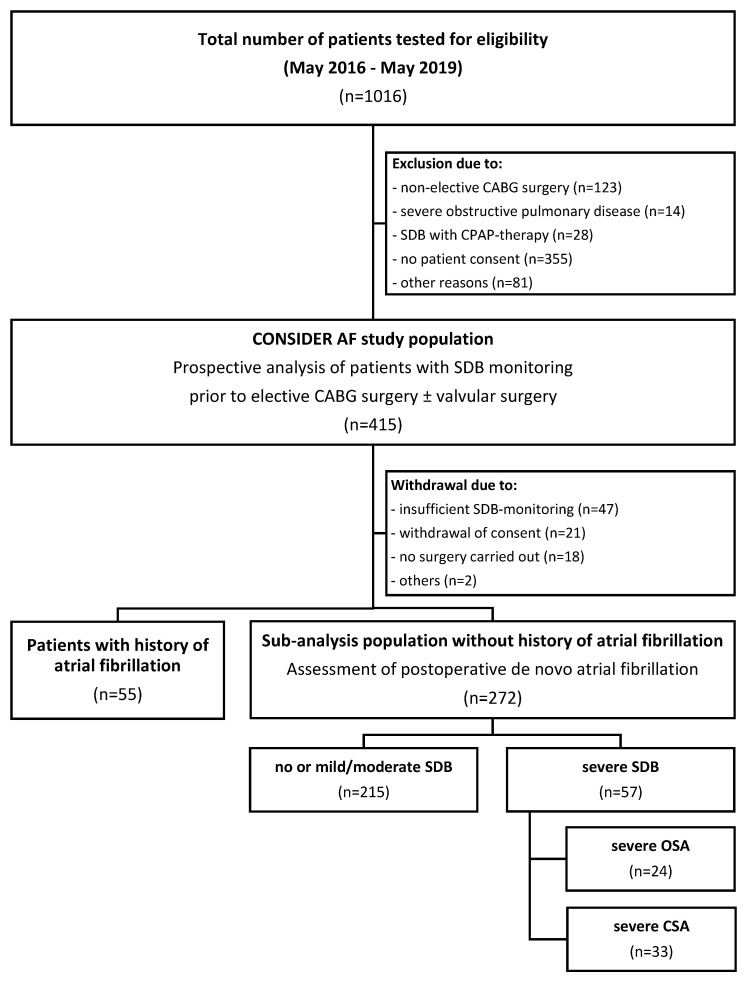
Study flow chart. CABG: coronary artery bypass grafting; SDB: sleep-disordered breathing; CPAP: continuous positive airway pressure; OSA: obstructive sleep apnea; CSA: central sleep apnea.

From May 2016 to May 2019, a collective of 415 patients were enrolled in the ongoing prospective observational study CONSIDER AF. Out of this cohort, 88 patients were excluded, primarily due to insufficient SDB monitoring, withdrawal of consent or short-term cancellation of CABG surgery (Figure 1). As 55 patients had a history of AF, they were omitted from the present sub-analysis of CONSIDER AF. Thus, the final sub-analysis cohort consisted of 272 patients who were classified according to the presence and type of severe SDB (Figure 1). The demographics of patients who were excluded or had a history of AF are summarized in Tables S1 and S2. 

The majority of participants were older men (Table 1). Previously undiagnosed severe SDB (AHI ≥ 30/h) was found in 21% of all patients, severe OSA in 9%, and severe CSA in 12% (Table 1 and Table S3). Please consult Table 1 for the prevalence rates of cardiovascular risk factors, additional comorbidities, laboratory findings, and preoperative data. Table 2 provides a summary of the data related to nocturnal respiration. Baseline and nocturnal respiration data according to the type of severe SDB or the presence of in-hospital POAF are shown in Table S3 and Table S4 or Table S5 and Table S6, respectively.

### 3.1. Postoperative De Novo Atrial Fibrillation and Potential Predictors

**Figure 2:**
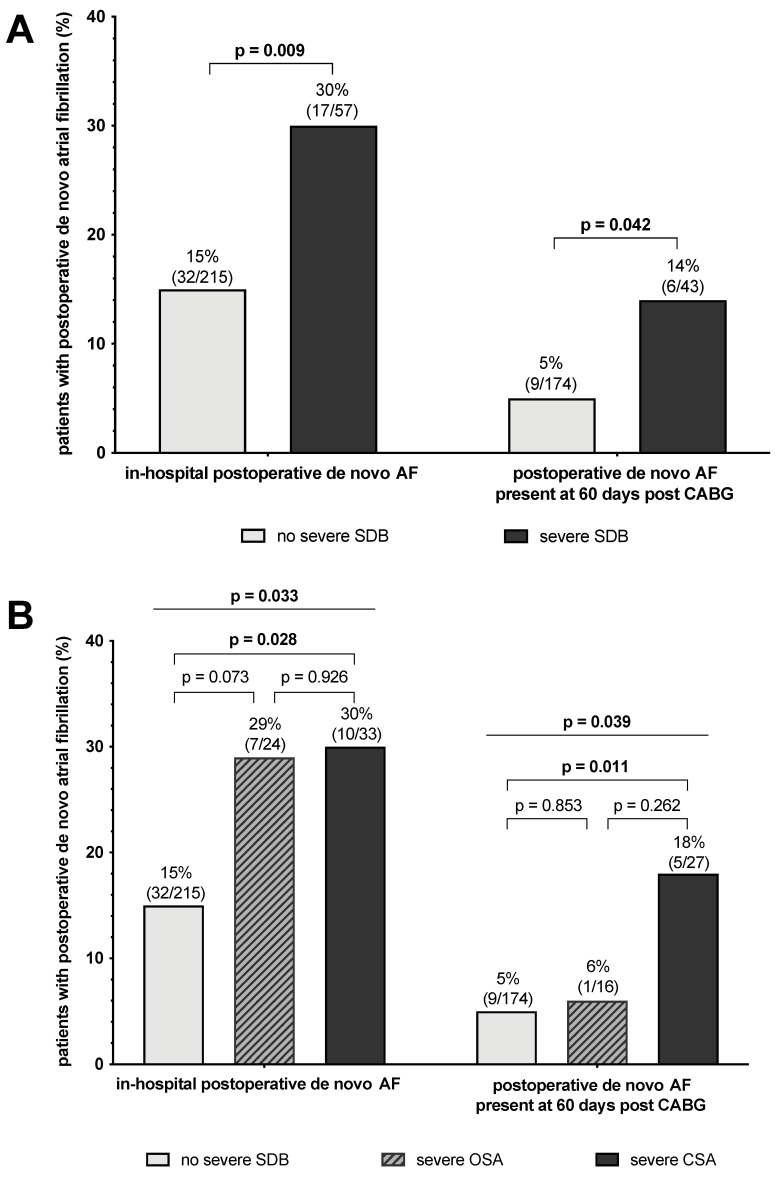
Incidence of in-hospital and late postoperative de novo atrial fibrillation. Incidence of in-hospital postoperative de novo atrial fibrillation and postoperative de novo atrial fibrillation that is still present at 60 days post CABG surgery with and without the presence of severe sleep-disordered breathing (**A**) as well as without severe sleep-disordered breathing, with severe obstructive and with severe central sleep-disordered breathing (**B**). Data are presented as percentages of patients with postoperative de novo atrial fibrillation in each group. SDB: sleep-disordered breathing; OSA: obstructive sleep apnea; CSA: central sleep apnea; AF: atrial fibrillation; CABG: coronary artery bypass grafting.

In-hospital POAF was diagnosed in 18% of patients (n = 49). In-hospital POAF was significantly more frequent in patients with severe SDB than in patients without severe SDB (30% vs. 15%, *p* = 0.009; Figure 2A). Rates of post-hospital POAF were low and similar among patients with and without severe SDB (2% vs. 3%, *p* = 0.844). Patients with severe SDB suffered significantly more often from recurrent AF episodes that were still present at 60 days post CABG surgery compared to patients without severe SDB (14% vs. 5%, *p* = 0.042; Figure 2A). 

The incidence of in-hospital POAF was similar in patients with severe CSA and those with severe OSA (Figure 2B). However, patients with severe CSA were affected three times more often by AF that was still present at 60 days post CABG than patients with severe OSA or without severe SDB (18% vs. 6% vs. 5%, *p* = 0.039; Figure 2B). The online data supplement offers further information on postoperative outcomes.

Severe SDB and established risk factors for POAF, such as advanced age ≥ 65 years, overweight, diabetes mellitus, a CHA_2_DS_2_-VASc-score of ≥5 points, renal failure, impaired left ventricular ejection fraction, and left atrial enlargement, were significantly associated with in-hospital POAF (Figure S1). Multivariable logistic regression analyses with severe SDB vs. no severe SDB and key demographic parameters showed that severe SDB and age ≥ 65 years were significantly associated with in-hospital POAF (Model I [demographic model], Table 3). Next to age ≥ 65 years and diabetes mellitus, the association between severe SDB and in-hospital POAF remained significant in an extended multivariable logistic regression model with established and clinically relevant confounders (Model II [full model], Table 3). When heart failure was added (Model III [heart failure model]), severe SDB was no longer significantly associated with in-hospital POAF (OR [95% CI]: severe SDB 1.99 [0.92; 4.31], *p*-value 0.081, Table 3). Please refer to the online data supplement for respective multivariable logistic regression analyses with CSA as an independent variable (Table S11).

### 3.2. Postoperative Outcome

The median length of hospital stay was 10 days for all patients (Tables S7 and S8). Compared to patients without in-hospital POAF, those with in-hospital POAF had a significantly longer hospital stay (median [25.; 75. percentile]: 9 [7; 12] vs. 14 [8; 18] days, *p* < 0.001) and longer time spent in the intensive care unit and in intermediate care (4 [2; 6] vs. 6 [2; 9] days, *p* = 0.003; Table S8).

Patients with in-hospital POAF experienced significantly more frequent hemodynamic complications, such as acute heart failure, a prolonged need for catecholamines, and postoperative hypoxemia, than patients without in-hospital POAF (Table S8). Acute kidney injury stages 2–3 and delirium occurred significantly more often in patients with in-hospital POAF than in patients without in-hospital POAF (Table S8). The use of pre- and postoperative diuretics was similar in patients with and without in-hospital POAF (Table S9). In the subgroup of patients with severe CSA, postoperative use of Spironolactone was significantly greater than those without severe SDB (Table S10). Information on preoperative medication in patients without severe SDB, with severe OSA and with severe CSA as well as in patients with and without POAF can be found in the online data supplement (Tables S13 and S14).

## 4. Discussion

This present sub-analysis of CONSIDER AF provides the following novel findings: Firstly, patients undergoing CABG surgery with severe SDB were significantly more often affected by in-hospital POAF and by POAF that was still present at 60 days post CABG surgery than patients without severe SDB. Secondly, severe SDB alongside advanced age and diabetes mellitus was significantly associated with in-hospital POAF after adjustment for known confounders. Thirdly, in patients with in-hospital POAF, the length of hospital stay and time spent in the ICU and IMC were significantly longer compared to those without in-hospital POAF.

### 4.1. Postoperative De Novo Atrial Fibrillation

Overall, in-hospital POAF was detected in 18% of our patients. Our findings are in line with previous studies on patients undergoing cardiac surgery that have described in-hospital POAF in 10% to 65% of patients [26,27]. Despite major advances in surgical and anesthetic techniques and general improvements in cardiac surgery-related outcomes, the incidence of POAF has remained unchanged over the last few decades [2,28,29]. The incidence of POAF fluctuates based on the patient characteristics and pre-existing comorbidities (e.g., age and severity of heart disease) as well as the type of surgery (e.g., isolated CABG surgery or additional valve surgery) [26,27]. Moreover, discrepancies in incidence rates of POAF may be due to diverse diagnostic modalities (e.g., 12-lead resting ECG or continuous telemetry-ECG monitoring) and study methodologies [27]. 

Based on the current literature, more than 90% of de novo POAF is assumed to resolve within 4–6 weeks postoperatively [26,27]. Therefore, most studies on POAF in patients undergoing cardiac surgery merely focus on in-hospital POAF. Notably, we found that one-third of patients with in-hospital POAF suffered from AF that was still present at 60 days post CABG surgery as detected by routinely performed Holter-ECG monitoring, which underlines the clinical relevance of POAF.

In the present study, severe SDB was significantly associated with a more than 2-fold increased risk of in-hospital POAF after elective CABG surgery. Regarding SDB subtypes, the incidence rates for in-hospital POAF were the same in both patient groups with severe OSA and severe CSA. When heart failure was added (Model III [heart failure model]), the odds ratio for severe SDB to predict in-hospital POAF was still 2-fold increased, although this association was no longer statistically significant. In the context of 49 in-hospital POAF cases and multiple adjustments in model III (heart failure model), we cannot rule out overfitting and type II errors. Therefore, further studies are needed to evaluate whether SDB contributes to POAF independently of heart failure.

All preceding studies on this topic have exclusively concentrated on patients both with and without OSA or SDB (see Table S12). In a retrospective study by Patel et al. [20], which was consistent with our findings, high risk for OSA was significantly associated with 2.9 greater odds for in-hospital POAF. Similarly, van Oosten et al. reported that the risk for in-hospital POAF was elevated 2.2-fold in patients with high risk for OSA [18]. However, there was no standardized SDB monitoring prior to CABG surgery in either study. High risk for OSA was rather mainly based on an unvalidated combination of clinical criteria [20] or on the Berlin questionnaire [18]. Unlike Patel et al., van Oosten et al. did not account for relevant comorbidities in multivariable regression analyses. Therefore, the independent risk for in-hospital POAF that can be attributable to high risk for OSA remains unclear [18]. As neither study monitored patients beyond their discharge date, there is no information available regarding post-hospital POAF or their long-term outcomes. The only study that performed standard polysomnography prior to CABG surgery found no significant difference in in-hospital POAF between patients with and without OSA, while POAF was significantly more common in patients with OSA compared to patients without OSA in the long-term follow-up [10]. Yet, this study by Uchôa et al. is limited by its small population, the lack of multivariable adjustments, and the fact that CSA was not taken into account [10].

When CSA is assessed, patients with SDB undergoing CABG surgery have a high proportion of CSA (range 25% to 27%) [23,30,31] since approximately one-third of these patients suffer from heart failure [21]. Remarkably, CSA has never been evaluated as a risk factor for POAF so far, although existing data suggest that central events are strongly associated with atrial arrhythmogenesis [32] and CSA predicted incident AF in an elderly patient cohort after accounting for confounders [33]. Our findings also indicate that patients with CSA may be affected more frequently by recurrent AF episodes that are still present at 60 days post CABG than patients with severe OSA or without severe SDB, even though this trend was not statistically significant. To this point, our data can merely be hypotheses-generating, and we cannot exclude the possibility that CSA only constitutes a surrogate marker of the underlying heart disease that causes POAF [34]. 

The development of POAF after cardiac surgery is of multifactorial origin and is triggered by patient, surgical, anesthetic, and postoperative factors [4]. Focusing on the association between SDB and AF, several pathophysiological mechanisms have been identified: 

Patients with OSA are exposed to intermittent hypoxia, hypercapnia, and activation of the sympathetic nervous system due to arousal episodes during sleep that may all predispose patients to AF [35,36]. Moreover, an increase in nocturnal arterial blood pressure and forceful ventilatory efforts during obstructive apneas result in negative intrathoracic pressure swings and increased transmural wall stress. Subsequently, OSA contributes to the dilation of the atria and triggers stretch-activated atrial ion channels. Recently, we showed that OSA oxidizes and activates a pivotal stress kinase, Ca/Calmodulin kinase II, leading to enhanced late Na current and pro-arrhythmic activity in the atrial tissue of patients subjected to cardio surgery [37]. These alterations may ultimately increase the risk for AF [20,35,36,38,39]. Beyond that, diagnosis of OSA has also been linked to electrical abnormalities (e.g., longer P-wave duration, prolonged conduction times, and longer sinus node recovery) that may render patients susceptible to developing AF [40].

Less is known about the interaction between CSA and atrial fibrillation. In a prospective study by Fox et al. investigating the relationship between restoration of sinus rhythm and SDB in patients with AF or atrial flutter, an immediate reduction in SDB was detected after cardioversion due to a significant reduction in central respiratory events [41]. Although the association between CSA and AF was reported to be independent of heart failure [33,42], it is important to interpret the findings within the context of a bidirectional causal relationship between CSA and heart failure [43,44]. Thus, CSA may only reflect the severity of heart failure, which contributes to CSA as a common manifestation of respiratory control instability via overnight rostral fluid displacement to the lungs and is itself well known to be related to AF [41,45]. Conversely, sleep apnea has the potential to worsen heart failure through various mechanisms. It exposes the heart to intermittent hypoxia, which can lead to oxygen deprivation. Furthermore, it elevates both preload and afterload on the heart, intensifying its workload. Additionally, sleep apnea increases sympathetic activity and impairs vascular endothelial function, compounding its detrimental effects on heart failure [43]. Next to left ventricular impairment, right ventricular dysfunction is also a strong predictor for AF. Pulmonary arterial hypertension and right ventricular dysfunction are common in patients with CSA and, thus, may predispose the development of AF [45].

### 4.2. Postoperative Outcome 

In the present study, patients with in-hospital POAF required prolonged postoperative in-patient treatment. The presence of in-hospital POAF extended the time spent in the ICU/IMC by 2 days and the total duration of hospitalization by 5 days compared to patients without in-hospital POAF. Multiple studies have consistently shown that patients who develop in-hospital POAF require approximately 24 h prolonged ICU time and an additional 2–5 days in hospital [6,20,26,46,47].

Due to the prolongation of hospitalization, in-hospital POAF ties up substantial financial resources. POAF was reported to entail approximately USD 10,000–20,000 in additional hospital costs. Beyond that, healthcare expenditures related to the burden of POAF in the United States were estimated at over USD 1 billion annually [6,29]. 

### 4.3. Clinical Implication

Our findings support recommendations to raise awareness for potential risk factors for POAF and systematically screen all patients who are scheduled for CABG surgery for diabetes mellitus and SDB within their routine preoperative risk assessment, even in the absence of indicative symptoms. According to current guidelines on SDB and perioperative management [48,49], all patients should preoperatively be assessed for their risk of SDB using validated questionnaires, such as the Berlin Questionnaire or the STOP-Bang questionnaire. Elective patients with a high pre-test probability of SDB should undergo thorough SDB monitoring before surgery [49]. Initiation of continuous positive airway pressure (CPAP) therapy prior to surgery should be considered in patients diagnosed with SDB; continuation of CPAP therapy in the immediate postoperative period should be encouraged for patients already on CPAP therapy [49].

However, as these recommendations are not based on randomized controlled trials, large-scale randomized controlled clinical studies are needed to evaluate whether preoperative screening for SDB and initiation of adequate SDB treatment may alleviate the risk for PAOF and other perioperative complications in patients undergoing cardiac surgery; whether this may be an effective approach to shorten postoperative length of hospitalization and eventually save in-hospital treatment costs warrants further investigation.

### 4.4. Limitations

Although polysomnography is recognized as the gold standard for diagnosing SDB, this study employed a portable SDB-monitoring device. This methodological compromise was considered the best option with regard to feasibility, access to a representative patient population, and validity of the CSA/OSA classification. Moreover, the present observational study was designed to assess association, but it cannot prove a causal relationship between severe SDB and POAF. 

Silent AF is reported to be a strong predictor of POAF. However, we did not systematically screen patients for silent AF prior to CABG surgery. Preoperative diagnosis of AF was solely based on the patients’ medical histories and a 12-lead resting ECG performed preoperatively within the routine preparation for cardiac surgery. Without preoperative screening for silent AF, some patients who already have undetected and asymptomatic paroxysmal AF may be misclassified as having de novo AF postoperatively. This misclassification could possibly lead to an overestimation of the incidence of de novo AF following CABG surgery and misinterpretation of confounding risk factors for POAF. Similarly, outcomes associated with postoperative AF may be inaccurately attributed to de novo POAF rather than the continuation or exacerbation of pre-existing AF. To mitigate these issues, future studies should include comprehensive screening for silent AF, for example, by using continuous ECG Holter monitoring preoperatively. Additionally, we evaluated in-hospital and post-hospital POAF in a merely binary fashion (present or absent) and did not investigate AF burden. 

Given the single-center design of our study, the generalizability of our findings is limited. However, this limitation underscores the need for further multi-center randomized controlled clinical trials.

## 5. Conclusions

Our findings indicate that in-hospital POAF affects approximately one-fifth of patients undergoing CABG surgery. Amongst established risk factors for POAF, such as advanced age and diabetes mellitus, which were tested in multivariable logistic regression analysis, severe SDB was associated with in-hospital POAF. Whether SDB and, in particular, CSA contribute to the development of POAF after cardiac surgery that may be alleviated by proper treatment merits further investigation.

## Figures and Tables

**Table 1:** Patient characteristics.

	Total Sub-Analysis Cohort	No Severe SDB (AHI < 30/h)	Severe SDB (AHI ≥ 30/h)	*p*-Value
**Demographic data**				
n (%)	272 (100)	215 (79)	57 (21)	
Age, years	66.4 ± 8.5	66.2 ± 8.7	67.4 ± 7.8	0.359 ^T^
Male sex, n (%)	228 (84)	177 (82)	51 (89)	0.193 ^Chi^
Body mass index, kg/m^2^	28.7 ± 4.4	28.4 ± 4.2	29.8 ± 4.9	**0.032 ^T^**
**Cardiovascular risk factors**				
Hypertension, n (%)	231 (85)	179 (83)	52 (91)	0.135 ^Chi^
Hypercholesterinemia, n (%)	190 (70)	147 (68)	43 (75)	0.301 ^Chi^
Diabetes mellitus, n (%)	94 (35)	73 (34)	21 (37)	0.683 ^Chi^
Smoking, n (%)	181 (66)	137 (64)	44 (77)	0.055 ^Chi^
CHA_2_DS_2_-VASc score, points	5.0 ± 1.1	4.9 ± 1.0	5.4 ± 1.2	**0.008 ^T^**
**Comorbidities**				
Heart failure _GFR adjusted_ *, n (%)	37 (16)	22 (12)	15 (30)	**0.002 ^Chi^**
NYHA III/IV, n (%)	69 (25)	50 (23)	19 (33)	0.120 ^Chi^
History of myocardial infarction, n (%)	79 (29)	60 (28)	19 (33)	0.434 ^Chi^
History of TIA or stroke, n (%)	43 (16)	31 (14)	12 (21)	0.222 ^Chi^
Renal failure ^†^, n (%)	59 (22)	44 (21)	15 (27)	0.316 ^Chi^
Anemia ^‡^, n (%)	64 (24)	50 (23)	14 (25)	0.864 ^Chi^
COPD, n (%)	12 (4)	10 (5)	2 (3)	0.709 ^Chi^
Depression, n (%)	12 (4)	11 (5)	1 (2)	0.272 ^Chi^
**Echocardiography parameters**				
LV ejection fraction, %	55.8 ± 11.0	57.5 ± 10.3	49.6 ± 11.8	**<0.001 ^T^**
LV ejection fraction < 55%, n (%)	61 (24)	41 (21)	20 (39)	**0.006 ^Chi^**
Left atrial enlargement ≥ 20 cm^2^	92 (40)	70 (39)	22 (47)	0.311 ^Chi^
Structural heart disease	123 (51)	92 (48)	31(62)	0.082 ^Chi^
**Laboratory data**				
NT-proBNP, pg/mL	229 (86; 713)	168 (73; 577)	625 (171; 1515)	**<0.001 ^W^**
Hemoglobin, g/dL	14.1 (12.7; 15.0)	14.1 (12.7; 15.0)	13.8 (12.7; 14.9)	0.671 ^W^
Creatinine, mg/dL	0.9 (0.8; 1.1)	0.9 (0.8; 1.1)	1.1 (0.8; 1.2)	**0.032 ^W^**
GFR, mL/min/1,73 m^2^	80 (63; 91)	81 (64; 92)	74 (58; 88)	**0.048 ^W^**
HbA1c, %	5.8 (5.5; 6.5)	5.8 (5.5; 6.5)	5.9 (5.5; 6.7)	0.574 ^W^
**Preoperative data**				
CABG and valve replacement, n (%)	53 (19)	42 (19)	11 (19)	0.968 ^Chi^
Number of stenoses, n	4 (3; 5)	4 (3; 5)	4 (3; 5)	0.575 ^W^
Number of grafts, n	3 (2; 3)	3 (2; 3)	3 (2; 3)	0.816 ^W^

Baseline variables of the study population of patients (n = 272) without and with severe SDB. Data are presented as mean ± standard deviation, median (interquartile range), or absolute and relative frequencies. ^T^ Students *t*-test; ^Chi^ Chi-square test; ^W^ Mann–Whitney U test. SDB: sleep-disordered breathing; AHI: apnea–hypopnea index; NYHA: New York Heart Association; LV: left ventricular; TIA: transient ischemic attack; COPD: chronic obstructive pulmonary disease; NT-proBNP: N-terminal pro-brain natriuretic peptide; GFR: glomerular filtration rate; CABG: coronary artery bypass grafting. * n = 233; NT-proBNP ≥ 450 pg/mL (patients < 50 years of age), ≥900 pg/mL (patients ≥ 50 and <75 years of age) or ≥1800 pg/mL (patients ≥ 75 years of age); ^†^ glomerular filtration rate < 60 mL/min/1.73 m^2^; ^‡^ hemoglobin < 12 g/dL (women) or hemoglobin < 13 g/dL (men). Bold values denote statistical significance at the *p* < 0.05 level.

**Table 2:** Nocturnal respiration data.

	Total Sub-Analysis Cohort	No Severe SDB (AHI < 30/h)	Severe SDB (AHI ≥ 30/h)	*p*-Value
Total recording time, min	484 (464; 499)	484 (466; 499)	480 (460; 496)	0.144 ^W^
Apnea hypopnea index, per hour	14.3 (6.7; 26.4)	11.0 (5.7; 17.3)	41.3 (33.9; 52.8)	**<0.001 ^W^**
Obstructive apnea index, per hour	2.6 (0.9; 6.7)	2.1 (0.8; 4.4)	10.6 (3.1; 21.6)	**<0.001 ^W^**
Central apnea index, per hour	1.9 (0.5; 7.1)	1.4 (0.4; 4.5)	13.9 (3.0; 31.7)	**<0.001 ^W^**
Oxygen desaturation index, per hour	11.7 (5.4; 21.9)	8.5 (3.8; 14.6)	36.6 (30.8; 47.2)	**<0.001 ^W^**
Mean SpO_2_, %	92 (91; 93)	92 (91; 93)	92 (90; 93)	**0.005 ^W^**
Time of SpO_2_ < 90%/total recording time, %	7.8 (1.5; 22.5)	5.2 (0.7; 19.2)	16.9 (8.4; 43.6)	**<0.001 ^W^**
mean heart rate, per minute	69 (64; 76)	69 (64; 76)	71 (66; 76)	0.132 ^W^
maximum heart rate, per minute	93 (83; 107)	93 (83; 107)	94 (83; 104)	0.729 ^W^

Nocturnal respiration data of the study population of patients (n = 272) without and with severe SDB. Data are presented as median (interquartile range). ^W^ Mann–Whitney U test. SDB: sleep-disordered breathing; AHI: apnea–hypopnea index. Bold values denote statistical significance at the *p* < 0.05 level.

**Table 3:** Multivariable logistic regression for in-hospital postoperative de novo atrial fibrillation as dependent variable.

Independent Variable	Model I (Demographic Model) c-Index: 0.703			Model II (Full Model) c-Index: 0.723			Model III (Heart Failure Model) c-Index: 0.758		
	OR	95% CI	*p*-Value	OR	95% CI	*p*-Value	OR	95% CI	*p*-Value
severe SDB (reference: no or mild/moderate SDB)	2.23	(1.10; 4.51)	**0.025**	2.23	(1.08; 4.61)	**0.030**	1.99	(0.92; 4.31)	0.081
Age ≥ 65 years	2.41	(1.19; 4,89)	**0.014**	2.17	(1.04; 4.53)	**0.038**	2.39	(1.09; 5.23)	**0.030**
Male sex	3.00	(0.87; 10.37)	0.083	2.76	(0.79; 9.63)	0.112	2.81	(0.78; 10.10)	0.114
Body mass index ≥ 25 kg/m^2^	3.44	(0.99; 11.91)	0.051	2.94	(0.84; 10.27)	0.092	3.30	(0.92; 11.78)	0.066
Diabetes mellitus				2.27	(1.15; 4.48)	**0.018**	1.98	(0.98; 4.02)	0.057
Renal failure ^†^				1.36	(0.64; 2.91)	0.427	1.34	(0.61; 2.96)	0.466
Heart failure _GFR adjusted_ *							1.76	(0.78; 3.93)	0.171

Multivariable regression analysis. Association of SDB and other preoperative predictors with postoperative in-hospital de novo atrial fibrillation. Values are presented as OR: odds ratio and 95% CI: confidence interval; SDB: sleep-disordered breathing. ^†^ glomerular filtration rate < 60 mL/min/1.73 m^2^; * n = 249; NTpro-BNP (adjusted to GFR according to Luchner et al. Clin Chem Lab Med 2010) ≥ 450 pg/mL (patients < 50 years of age), ≥900 pg/mL (patients ≥ 50 and <75 years of age) or ≥1800 pg/mL (patients ≥ 75 years of age). Bold values denote statistical significance at the *p* < 0.05 level.

## Data Availability

Data are contained within the article and Supplementary Materials.

## Supplementary Materials

The following supporting information can be downloaded at: https://www.mdpi.com/article/10.3390/biomedicines12051035/s1, Table S1: Patient characteristics; Table S2: Patient characteristics; Table S3: Patient characteristics (SDB-groups); Table S4: Nocturnal respiration data (SDB-groups); Table S5: Patient characteristics (POAF-groups); Table S6: Nocturnal respiration data (POAF-groups); Table S7: Postoperative outcome (SDB-groups); Table S8: Postoperative outcome (POAF-groups); Table S9: Pre- and postoperative diuretics in patients with and without postoperative de-novo atrial fibrillation (POAF-groups); Table S10: Pre- and postoperative diuretics in patients without severe SDB, with severe OSA and with severe CSA (SDB-groups); Table S11: Multivariable logistic regression for in-hospital postoperative de-novo atrial fibrillation as dependent variable; Table S12: Studies addressing the association between sleep-disordered breathing and postoperative de-novo atrial fibrillation after cardiac surgery; Table S13: Preoperative medication in patients without severe SDB, with severe OSA and with severe CSA (SDB-groups); Table S14: Preoperative medication in patients with and without postoperative de-novo atrial fibrillation (POAF-groups); Figure S1: Preoperative predictors of in-hospital postoperative de-novo atrial fibrillation.

## Abbreviations

AF	Atrial fibrillation
CABG	Coronary artery bypass grafting
CI	Confidence interval
CSA	Central sleep apnoea
ICU	Intensive care unit
IMC	Intermediate care
LOS	Length of hospital stay
OR	Odds ratio
OSA	Obstructive sleep apnoea
POAF	Postoperative de novo atrial fibrillation
SDB	Sleep-disordered breathing
