# Supplementary material for: Sleep-Disordered Breathing, Advanced Age, and Diabetes Mellitus Are Associated with De Novo Atrial Fibrillation after Cardiac Surgery
Source: Biomedicines. 2024 May 8;12(5):1035. doi: 10.3390/biomedicines12051035 (PMC11117485; doi:10.3390/biomedicines12051035)
Supplement: Supplementary file 1 [file biomedicines-12-01035-s001.zip › biomedicines-2937288-supplementary.pdf]

Sleep-Disordered Breathing, Advanced Age, and Diabetes Mellitus Are Associated with De Novo Atrial Fibrillation after Cardiac Surgery

- Online data supplement -

Supplemental methods

Assessment of sleep-disordered breathing

As described previously, apnea was defined as a ≥90 decrease in airflow for ≥10 seconds, hypopnea as a decrease in airflow by ≥30-90% versus baseline for ≥10 seconds, and desaturation as a ≥4% decrease in oxygen saturation. The apnea-hypopnea index (AHI) is expressed as the frequency of apnea or hypopnea events per hour recording time, and an AHI of ≥15/hour was considered the cut-off for the diagnosis of SDB. Patients with SDB and ≥50% of central apnea events were classified into the CSA group and patients with <50% of central apnea events into the OSA group.

Standardized clinical treatment

In general, all patients undergoing elective CABG surgery at the Department of Cardiothoracic Surgery of the University Medical Center Regensburg (Germany) get admitted to the hospital one day prior to CABG surgery. No premedication was routinely administered the night prior to surgery.

Anesthesia was induced with a standardized regimen of sufentanil, etomidate, and rocuronium and maintained with sufentanil and sevoflurane. Details of the maintenance of anesthesia were at the discretion of the anesthesiologist. After surgery, all patients were transferred to the intensive care unit (ICU) while being intubated and sedated. Standardized postoperative sedation consisted of propofol and opioids.

All patients received postoperative treatment according to the 'fast-track recovery' care protocol that aims at early extubation, prompt implementation of mobility, and occupational therapy within ICU and intermediate care (IMC) settings. Patients were weaned from mechanical ventilation at the discretion of the ICU physicians. All patients received supplemental oxygen according to the standards of the surgical ICU and IMC, but not with the intention to treat sleep-disordered breathing. Within the standard postoperative care of patients, special emphasis was placed on the rational titration of sedatives and opioids, reduced exposure to benzodiazepines, and appropriate use of antipsychotic drugs.

To reduce bias, portable SDB-monitoring and perioperative clinical treatment were performed in a standardized manner (e.g. 'fast-track recovery' care protocol; online data supplement). Medical staff, including nurses, anesthesiologists and intensive care unit (ICU) physicians as well as physiotherapists, were blinded to the patients' diagnosis of SDB. Thus, none of the patients received specific treatment for sleep-disordered breathing in the perioperative phase.

Assessment of postoperative outcome

For assessment of postoperative outcome, hemodynamic, respiratory, renal and neurological complications were analyzed with regard to the presence of severe SDB and to the presence of in-hospital postoperative de-novo AF. Moreover, length of hospital stay (LOS) and time spent in the ICU/intermediate care (IMC) were evaluated. LOS - classified as the time between surgery and the day of discharge - was considered prolonged, when it was above the median length of hospitalization of all patients.

Supplemental results

Postoperative outcome

The median LOS was 10 days for all patients (e-Table S7 and e-Table S8). Compared to patients without in-hospital POAF those with in-hospital POAF had a significantly longer hospital stay (median [25.; 75. percentile]: 9 [7; 12] vs. 14 [8; 18] days, $p<0.001$) and longer time spent in the ICU and IMC (4 [2; 6] vs. 6 [2; 9] days, $p=0.003$; e-Table S7). Patients with in-hospital POAF experienced significantly more often hemodynamic complications, such as acute heart failure and a prolonged need for catecholamines, and postoperative hypoxemia than patients without in-hospital POAF (e-Table S8). Acute kidney injury stages 2-3 and delirium occurred significantly more often in patients with in-hospital POAF than in patients without in-hospital POAF (e-Table S8). Use of pre- and postoperative diuretics was similar in patients with and without in-hospital POAF (e-Table S9). In the subgroup of patients with severe CSA postoperative use of Spironolactone was significantly greater compared to those without severe SDB (e-Table S10).

Supplementary Tables

Table S1 Patient characteristics

	**overall**	**sub-analysis population**	**drop-out population (total)**	**p-value**
n (%)	415	272 (100)	143 (100)	
Age, years	67.4 ± 8.5	66.4 ± 8.5	69.4 ± 8.0	**<0.001 T**
Male sex, n (%)	353 (85)	228 (84)	123 (86)	0.647 Chi
Body mass index, kg/m²	28.7 ± 4.7	28.7 ± 4.4	28.7 ± 5.4	0.961 T
Impaired LV ejection fraction < 55%	94 (28)	61 (24)	33 (41)	**0.005 Chi**
NT-proBNP, pg/ml	337 (99; 1103)	229 (86; 713) *	1158 (495; 2560) †	**<0.001 W**

Baseline variables of the sub-analysis population and of patients who were excluded or had a history of atrial fibrillation (drop-out population). Data are presented as mean ± standard deviation or median (interquartile range) unless otherwise stated. NT-pro BNP: N-terminal pro-brain natriuretic peptide; * n=233; † n=63.

Table S2 Patient characteristics

	**overall**	**sub-analysis population**	**withdrawal population**	**history of AF population**	**p-value**
n (%)	415	272 (100)	88 (100)	55 (100)	
Age, years	67.4 ± 8.5	66.4 ± 8.5 b	68.7 ± 8.7	70.4 ± 6.9 b	**0.002 A**
Male sex, n (%)	353 (85)	228 (84)	73 (84)	49 (89)	0.628 Chi
Body mass index, kg/m²	28.7 ± 4.7	28.7 ± 4.4	28.4 ± 6.3	29.1 ± 4.2	0.729 A
Impaired LV ejection fraction < 55%	94 (28)	61 (24) b	10 (36)	23 (43) b	**0.015 Chi**
NT-proBNP, pg/ml	337 (99; 1103)	229 (86; 713) * a b	785 (337; 7575) a	1167 (495; 2509) b	**<0.001 KW**

Baseline variables of the sub-analysis population and of patients who were excluded or had a history of atrial fibrillation (drop-out population). Data are presented as mean ± standard deviation or median (interquartile range) unless otherwise stated. NT-pro BNP: N-terminal pro-brain natriuretic peptide. [A] Anova test; [Chi] Chi-square test; [KW] Kruskal-Wallis test. [a] $p_{\text{sub-analysis vs. withdrawal}}$ <0.05; [b] $p_{\text{sub-analysis vs. history of AF}}$ <0.05; [c] $p_{\text{withdrawal vs. history of AF}}$ <0.05.* n=233; † n=63.

Table S3 Patient characteristics (SDB-groups)

	total sub-analysis cohort	no severe SDB (AHI <30/h)	severe OSA (AHI ≥30/h)	severe CSA (AHI ≥30/h)	p-value
**Demographic data**					
n (%)	272 (100)	215 (79)	24 (9)	33 (12)	
Age, years	66.4 ± 8.5	66.2 ± 8.7	67.8 ± 7.7	67.1 ± 8.0	0.624 [A]
Male sex, n (%)	228 (84)	177 (82)	22 (92)	29 (88)	0.398 [Chi]
Body mass index, kg/m²	28.7 ± 4.4	28.4 ± 4.2 [a]	31.1 ± 5.0 [a]	28.8 ± 4.7	**0.016 [A]**
**Cardiovascular risk factors**					
Hypertension, n (%)	231 (85)	179 (83)	22 (92)	30 (91)	0.326 [Chi]
Hypercholesterinemia, n (%)	190 (70)	147 (68)	21 (87)	22 (67)	0.140 [Chi]
Diabetes mellitus, n (%)	94 (35)	73 (34)	7 (29)	14 (42)	0.536 [Chi]
Smoking, n (%)	181 (66)	137 (64)	19 (79)	25 (76)	0.154 [Chi]
CHA_2DS_2-VASc-Score, points	5.0 ± 1.1	4.9 ± 1.0 [b]	5.2 ± 1.2	5.5 ± 1.2 [b]	**0.021 [A]**
**Comorbidities**					
Heart failure $_{GFR\ adjusted}$*, n (%)	37 (16)	22 (12) [b]	3 (15)	12 (40) [b]	**0.001 [Chi]**
NYHA III/IV, n (%)	69 (25)	50 (23)	8 (33)	11 (33)	0.299 [Chi]
History of myocardial infarction, n (%)	79 (29)	60 (28)	6 (25)	13 (39)	0.367 [Chi]
History of TIA or stroke, n (%)	43 (16)	31 (14)	5 (21)	7 (21)	0.474 [Chi]
Renal failure†, n (%)	59 (22)	44 (21)	6 (26)	9 (27)	0.601 [Chi]
Anemia‡, n (%)	64 (24)	50 (23)	2 (8) [c]	12 (36) [c]	**0.048 [Chi]**
COPD, n (%)	12 (4)	10 (5)	1 (4)	1 (3)	0.913 [Chi]
Depression, n (%)	12 (4)	11 (5)	0 (0)	1 (3)	0.470 [Chi]
**Echocardiography parameters**					
LV ejection fraction, %	55.8 ± 11.0	57.5 ± 10.3 [b]	55.3 ± 6.1 [c]	45.5 ± 13.3 [b c]	**<0.001 [A]**
LV ejection fraction <55%, n (%)	61 (24)	41 (21) [b]	4 (18) [c]	16 (55) [b c]	**<0.001 [Chi]**
Left atrial enlargement ≥20 cm²	92 (40)	70 (39)	7 (32)	15 (60)	0.087 [Chi]
Structural heart disease	123 (51)	92 (48)	10 (45)	21 (75)	**0.026 [Chi]**
**Laboratory data**					
NT-proBNP, pg/ml	229 (86; 713)	168 (73; 577) [b]	312 (100; 1087)	921 (338; 1919) [b]	**<0.001 [KW]**
Hemoglobin, g/dl	14.1 (12.7; 15.0)	14.1 (12.7; 15.0)	14.0 (13.5; 14.7)	13.2 (12.3; 15.0)	0.462 [KW]
Creatinine, mg/dl	0.9 (0.8; 1.1)	0.9 (0.8; 1.1)	1.0 (0.8; 1.2)	1.1 (0.9; 1.2)	0.071 [KW]
GFR, ml/min/1,73 m²	80 (63; 91)	81 (64; 92)	78 (56; 90)	69 (58; 84)	0.104 [KW]
**Preoperative data**					
CABG and valve replacement, n (%)	53 (19)	42 (19)	3 (12)	8 (24)	0.543 [Chi]
Number of stenoses, n	4 (3; 5)	4 (3; 5)	4 (3; 5)	4 (3; 5)	0.652 [KW]
Number of grafts, n	3 (2; 3)	3 (2; 3)	3 (2; 3)	3 (2; 3)	0.867 [KW]

Baseline variables of the study population of patients (n=232) without severe SDB and with severe OSA and severe CSA. Data are presented as mean ± standard deviation, median (interquartile range) or absolute and relative frequencies. [A] Anova test; [Chi] Chi-square test; [KW] Kruskal-Wallis test. [a] $p_{severe\ OSA\ vs.\ no\ or\ mild/moderate\ SDB}$ <0.05; [b] $p_{severe\ CSA\ vs.\ no\ or\ mild/moderate\ SDB}$ <0.05; [c] $p_{severe\ OSA\ vs.\ severe\ CSA}$ <0.05. SDB: sleep-disordered breathing; AHI: apnea-hypopnea-index; OSA: obstructive sleep apnea; CSA: central sleep apnea; NYHA: New York Heart Association; LV: left ventricular; TIA: transient ischemic attack; COPD: chronic obstructive pulmonary disease; NT-proBNP: N-terminal pro-brain natriuretic peptide; GFR: glomerular filtration rate; CABG: coronary artery bypass grafting. * n=233; NT-proBNP ≥450 pg/mL (patients <50 years of age), ≥900 pg/mL (patients ≥50 and <75 years of age) or ≥1800 pg/mL (patients ≥75 years of age); † glomerular filtration rate <60 ml/min/1.73 m²; ‡ hemoglobin <12 g/dl (women) or hemoglobin <13 g/dl (men).

Table S4 Nocturnal respiration data (SDB-groups)

	total sub-analysis cohort	no severe SDB (AHI <30/h)	severe OSA (AHI ≥30/h)	severe CSA (AHI ≥30/h)	p-value
Total recording time, min	484 (464; 499)	484 (466; 499) [a]	466 (452; 483) [a c]	488 (464; 503) [c]	**0.006** [KW]
Apnea hypopnea index, per hour	14.3 (6.7; 26.4)	11.0 (5.7; 17.3) [a b]	39.9 (33.3; 51.5) [a]	41.7 (34.4; 54.4) [b]	**<0.001** [KW]
Obstructive apnea index, per hour	2.6 (0.9; 6.7)	2.1 (0.8; 4.4) [a b]	23.6 (14.7; 36.7) [a c]	5.2 (1.7; 10.1) [b c]	**<0.001** [KW]
Central apnea index, per hour	1.9 (0.5; 7.1)	1.4 (0.4; 4.5) [b]	2.3 (0.3; 8.2) [c]	28.4 (19.7; 37.1) [b c]	**<0.001** [KW]
Oxygen desaturation index, per hour	11.7 (5.4; 21.9)	8.5 (3.8; 14.6) [a b]	33.7 (30.0; 44.1) [a]	37.7 (31.8; 50.4) [b]	**<0.001** [KW]
Mean SpO_2, %	92 (91; 93)	92 (91; 93) [a]	91 (89; 93) [a]	92 (90; 93)	**0.009** [KW]
Time of SpO_2 <90%/total recording time, %	7.8 (1.5; 22.5)	5.2 (0.7; 19.2) [a b]	17.1 (9.7; 45.6) [a]	16.7 (6.3; 37.3) [b]	**<0.001** [KW]
mean heart rate, per minute	69 (64; 76)	69 (64; 76)	74 (68; 82)	69 (65; 76)	0.138 [KW]
maximum heart rate, per minute	93 (83; 107)	93 (83; 107)	95 (84; 104)	92 (81; 104)	0.817 [KW]

Nocturnal respiration data of the study population of patients (n=272) without severe SDB and with severe OSA and severe CSA. Data are presented as median (interquartile range). [KW] Kruskal-Wallis test. [a] $p_{\text{severe OSA vs. no or mild/moderate SDB}}$ <0.05; [b] $p_{\text{severe CSA vs. no or mild/moderate SDB}}$ <0.05; [c] $p_{\text{severe OSA vs. severe CSA}}$ <0.05. SDB: sleep-disordered breathing; AHI: apnea-hypopnea-index; OSA: obstructive sleep apnea; CSA: central sleep apnea.

Table S5 Patient characteristics (POAF-groups)

	total sub-analysis cohort	no in-hospital postoperative de-novo AF	in-hospital postoperative de-novo AF	p-value
**Demographic data**				
n (%)	272 (100)	223 (82)	49 (18)	
Age, years	66.4 ± 8.5	65.9 ± 8.7	68.8 ± 7.3	**0.028** [T]
Male sex, n (%)	228 (84)	182 (82)	46 (94)	**0.035** [Chi]
Body mass index, kg/m²	28.7 ± 4.4	28.5 ± 4.4	29.5 ± 4.6	0.154 [T]
**Cardiovascular risk factors**				
Hypertension, n (%)	231 (85)	188 (84)	43 (88)	0.541 [Chi]
Hypercholesterinemia, n (%)	190 (70)	159 (71)	31 (63)	0.267 [Chi]
Diabetes mellitus, n (%)	94 (35)	68 (30)	26 (53)	**0.003** [Chi]
Smoking, n (%)	181 (66)	145 (65)	36 (73)	0.257 [Chi]
CHA_2DS_2-VASc-Score, points	5.0 ± 1.1	4.9 ± 1.1	5.5 ± 1.0	**0.001** [Chi]
**Comorbidities**				
Heart failure $_{\text{GFR adjusted}}$*, n (%)	37 (16)	28 (15)	9 (22)	0.241 [Chi]
NYHA III/IV, n (%)	69 (25)	56 (25)	13 (26)	0.836 [Chi]
History of myocardial infarction, n (%)	79 (29)	68 (31)	11 (22)	0.254 [Chi]
History of TIA or stroke, n (%)	43 (16)	37 (17)	6 (12)	0.450 [Chi]
Renal failure†, n (%)	59 (22)	43 (19)	16 (33)	**0.043** [Chi]
Anemia‡, n (%)	64 (24)	51 (23)	13 (26)	0.607 [Chi]
COPD, n (%)	12 (4)	10 (4)	2 (4)	0.901 [Chi]
Depression, n (%)	12 (4)	11 (5)	1 (2)	0.372 [Chi]
**Echocardiography parameters**				
LV ejection fraction, %	55.8 ± 11.0	57.1 ± 9.9	49.4 ± 14.0	**0.013** [T]
LV ejection fraction <55%, n (%)	61 (24)	42 (21)	19 (40)	**0.005** [Chi]
Left atrial enlargement ≥20 cm^2	92 (40)	69 (37)	23 (55)	**0.035** [Chi]
Structural heart disease	123 (51)	95 (49)	28 (61)	0.138 [Chi]
**Laboratory data**				
NT-proBNP, pg/ml	229 (86; 713)	178 (80; 623)	570 (155; 1172)	**0.009** [W]
Hemoglobin, g/dl	14.1 (12.7; 15.0)	14.1 (12.7; 15.0)	13.9 (12.9; 14.4)	0.383 [W]
Creatinine, mg/dl	0.9 (0.8; 1.1)	0.9 (0.8; 1.1)	1.0 (0.8; 1.3)	0.074 [W]
GFR, ml/min/1,73 m^2	80 (63; 91)	81 (64; 92)	74 (54; 88)	**0.032** [W]
**Preoperative data**				
CABG and valve replacement, n (%)	53 (19)	43 (19)	10 (20)	0.857 [Chi]
Number of stenoses, n	4 (3; 5)	4 (3; 5)	4 (3; 5)	0.772 [W]
Number of grafts, n	3 (2; 3)	3 (2; 3)	3 (2; 3)	0.461 [W]

Baseline variables of the study population of patients (n=232) without and with in-hospital postoperative de-novo atrial fibrillation. Data are presented as mean ± standard deviation, median (interquartile range) or absolute and relative frequencies. [T] Students t-test; [Chi] Chi-square test; [W] Mann-Whitney-U-test. AF: atrial fibrillation; NYHA: New York Heart Association; LV: left ventricular; TIA: transient ischemic attack; COPD: chronic obstructive pulmonary disease; NT-proBNP: N-terminal pro-brain natriuretic peptide; GFR: glomerular filtration rate; CABG: coronary artery bypass grafting. * n=233; NT-proBNP ≥450 pg/mL (patients <50 years of age), ≥900 pg/mL (patients ≥50 and <75 years of age) or ≥1800 pg/mL (patients ≥75 years of age); † glomerular filtration rate <60 ml/min/1.73 m^2; ‡ hemoglobin <12 g/dl (women) or hemoglobin <13 g/dl (men).

Table S6 Nocturnal respiration data (POAF-groups)

	total sub-analysis cohort	no in-hospital postoperative de-novo AF	in-hospital postoperative de-novo AF	p-value
Total recording time, min	484 (464; 499)	484 (465; 500)	477 (461; 489)	0.090 [W]
Apnea hypopnea index, per hour	14.3 (6.7; 26.4)	14.1 (6.6; 24.4)	18.9 (8.1; 33.9)	0.090 [W]
Obstructive apnea index, per hour	2.6 (0.9; 6.7)	2.6 (0.9; 6.8)	3.3 (1.3; 6.3)	0.392 [W]
Central apnea index, per hour	1.9 (0.5; 7.1)	1.9 (0.5; 6.8)	1.4 (0.5; 10.4)	0.763 [W]
Oxygen desaturation index, per hour	11.7 (5.4; 21.9)	10.9 (5.0; 22.5)	14.6 (6.6; 32.2)	0.091 [W]
Mean SpO_2, %	92 (91; 93)	92 (91; 93)	92 (91; 93)	0.998 [W]
Time of SpO_2 <90%/total recording time, %	7.8 (1.5; 22.5)	7.4 (1.2; 21.7)	8.1 (3.0; 28.3)	0.285 [W]
mean heart rate, per minute	69 (64; 76)	69 (64; 76)	71 (64; 77)	0.907 [W]
maximum heart rate, per minute	93 (83; 107)	93 (83; 107)	95 (83; 106)	0.857 [W]
Sleep-disordered breathing (AHI ≥30/h), n (%)	57 (21)	40 (18)	17 (35)	**0.009** [Chi]
Obstructive sleep apnea (AHI ≥30/h), n (%)	24 (10)	17 (9)	7 (18)	0.073 [Chi]
Central sleep apnea (AHI ≥30/h), n (%)	33 (13)	23 (11)	10 (24)	**0.028** [Chi]

Nocturnal respiration data of the study population of patients (n=272) without and with in-hospital postoperative de-novo atrial fibrillation. Data are presented as median (interquartile range). [W] Mann-Whitney-U-test. AF: atrial fibrillation; AHI: apnea hypopnea index.

Table S7 Postoperative outcome (SDB-groups)

	total sub-analysis cohort	no severe SDB (AHI <30/h)	severe OSA (AHI ≥30/h)	severe CSA (AHI ≥30/h)	p-value
**Hemodynamic complications**					
Acute heart failure, n (%)	8 (3)	8 (4)	0 (0)	0 (0)	0.650 [Chi]
Need for catecholamines, hours	20 (12; 42)	19 (11; 40)	25 (10; 49)	26 (17; 70)	0.347 [KW]
Prolonged need for catecholamines ≥48 h, n (%)	51 (20)	33 (16)[b]	6 (25)	12 (39)[b]	**0.011 [Chi]**
Norepinephrine, n (%)	123 (47)	95 (46)	13 (54)	15 (48)	0.762 [Chi]
Epinephrine, n (%)	23 (9)	15 (7)[b]	1 (4)	7 (23)[b]	**0.014 [Chi]**
Dobutamine, n (%)	114 (44)	95 (46)	10 (42)	9 (29)	0.189 [Chi]
**Respiratory complications**					
Duration of invasive ventilation, hours	10 (7; 13)	10 (7; 13)	9 (7; 14)	11 (9; 14)	0.376 [KW]
Need for non-invasive ventilation, n (%)	40 (15)	32 (15)	3 (13)	5 (15)	0.949 [Chi]
(Non-) invasive ventilation >24 hours, n (%)	12 (30)	8 (25)	1 (33)	3 (60)	0.281 [Chi]
Postoperative hypoxemia, n (%)	80 (29)	62 (29)	6 (25)	12 (36)	0.604 [Chi]
Postoperative respiratory failure, n (%)	35 (13)	28 (13)	3 (13)	4 (12)	0.988 [Chi]
Reintubation, n (%)	11 (4)	9 (4)	1 (4)	1 (3)	0.950 [Chi]
Tracheotomy, n (%)	4 (1)	4 (2)	0 (0)	0 (0)	0.584 [Chi]
ECMO therapy, n (%)	4 (1)	3 (1)	0 (0)	1 (3)	0.631 [Chi]
**Renal and neurological complications**					
Acute kidney injury stage 2-3, n (%)	28 (10)	22 (10)	3 (12)	3 (9)	0.914 [Chi]
Delirium, n (%)	32 (12)	26 (12)	4 (17)	2 (6)	0.444 [Chi]
**Length of postoperative hospital stay**					
Length of hospital stay, days	10 (7; 14)	9 (7; 13)	11 (7; 15)	11 (8; 19)	0.367 [KW]
Length of ICU-/IMC stay, days	4 (2; 7)	4 (2; 6)	4 (2; 9)	5 (3; 8)	0.273 [KW]
Prolonged length of hospital stay >10 days, n (%)	122 (45)	93 (43)	12 (50)	17 (52)	0.585 [Chi]
**Medication at hospital discharge**					
Betablockers, n (%)	220 (82)	173 (82)	18 (75)	29 (88)	0.455 [Chi]
ACE inhibitors, n (%)	148 (55)	121 (57)	11 (46)	16 (48)	0.397 [Chi]
AT1 receptor antagonists, n (%)	46 (17)	32 (15)	5 (21)	9 (28)	0.178 [Chi]
Angiotensine receptor-neprilysin inhibitors, n (%)	2 (1)	2 (1)	0 (0)	0 (0)	0.759 [Chi]
Calcium channel blockers, n (%)	73 (27)	59 (28)	8 (33)	6 (19)	0.429 [Chi]
Antiarrhythmic agents, n (%)	4 (1)	3 (1)	1 (4)	0 (0)	0.441 [Chi]
Anticoagulants, n	69 (26)	46 (22)[b]	8 (33)	15 (46)[b]	**0.012 [Chi]**

Postoperative outcome of the study population of patients (n=272) without and with severe sleep-disordered breathing. Data are presented as median (interquartile range) or absolute and relative frequencies. [Chi] Chi-square test; [KW] Kruskal-Wallis test. [a] $p_{\text{severe OSA vs. no severe SDB}} < 0.05$; [b] $p_{\text{severe CSA vs. no severe SDB}} < 0.05$; [c] $p_{\text{severe OSA vs. severe CSA}} < 0.05$. AF: atrial fibrillation; ECMO: extracorporeal membrane oxygenation; ICU: intensive care unit; IMC: intermediate care.

Table S8 Postoperative outcome (POAF-groups)

	total sub-analysis cohort	no in-hospital postoperative de-novo AF	in-hospital postoperative de-novo AF	p-value
**Hemodynamic complications**				
Acute heart failure, n (%)	8 (3)	5 (2)	3 (6)	**0.034 Chi**
Need for catecholamines, hours	20 (12; 42)	19 (12; 38)	32 (11; 68)	**0.032 W**
Prolonged need for catecholamines ≥48 h, n (%)	51 (20)	33 (16)	18 (38)	**<0.001 Chi**
Norepinephrine, n (%)	123 (47)	102 (48)	21 (44)	0.585 Chi
Epinephrine, n (%)	23 (9)	15 (7)	8 (17)	**0.035 Chi**
Dobutamine, n (%)	114 (44)	95 (45)	19 (40)	0.510 Chi
**Respiratory complications**				
Duration of invasive ventilation, hours	10 (7; 13)	10 (7; 13)	11 (7; 16)	0.187 W
Need for non-invasive ventilation, n (%)	40 (15)	29 (13)	11 (22)	0.091 Chi
(Non-) invasive ventilation >24 hours, n (%)	12 (30)	7 (24)	5 (45)	0.189 Chi
Postoperative hypoxemia, n (%)	80 (29)	57 (26)	23 (47)	**0.003 Chi**
Postoperative respiratory failure, n (%)	35 (13)	26 (12)	9 (18)	0.204 Chi
Reintubation, n (%)	11 (4)	7 (3)	4 (8)	0.108 Chi
Tracheotomy, n (%)	4 (1)	3 (1)	1 (2)	0.714 Chi
ECMO therapy, n (%)	4 (1)	3 (1)	1 (2)	0.714 Chi
**Renal and neurological complications**				
Acute kidney injury stage 2-3, n (%)	28 (10)	18 (8)	10 (20)	**0.010 Chi**
Delirium, n (%)	32 (12)	20 (9)	12 (25)	**0.002 Chi**
**Length of postoperative hospital stay**				
Length of hospital stay, days	10 (7; 14)	9 (7; 12)	14 (8; 18)	**<0.001 W**
Length of ICU-/IMC stay, days	4 (2; 7)	4 (2; 6)	6 (2; 9)	**0.003 W**
Prolonged length of hospital stay >10 days, n (%)	122 (45)	90 (40)	32 (65)	**0.002 Chi**
**Medication at hospital discharge**				
Betablockers, n (%)	220 (82)	179 (82)	41(84)	0.749 Chi
ACE inhibitors, n (%)	148 (55)	123 (56)	25 (51)	0.513 Chi
AT1 receptor antagonists, n (%)	46 (17)	41 (19)	5 (10)	0.146 Chi
Angiotensine receptor-neprilysin inhibitors, n (%)	2 (1)	2 (1)	0 (0)	0.499 Chi
Calcium channel blockers, n (%)	73 (27)	57 (26)	16 (33)	0.376 Chi
Antiarrhythmic agents, n (%)	4 (1)	0 (0)	4 (8)	**<0.001 Chi**
Anticoagulants, n	69 (26)	51 (24)	18 (37)	0.059 Chi

Postoperative outcome of the study population of patients (n=272) without and with in-hospital postoperative de-novo atrial fibrillation. Data are presented as median (interquartile range) or absolute and relative frequencies. Chi Chi-square test; W Mann-Whitney-U-test. AF: atrial fibrillation; ECMO: extracorporeal membrane oxygenation; ICU: intensive care unit; IMC: intermediate care.

Table S9 Pre- and postoperative diuretics in patients with and without postoperative de-novo atrial fibrillation (POAF-groups)

	total sub-analysis cohort	no in-hospital postoperative de-novo AF	in-hospital postoperative de-novo AF	p-value
**Preoperative diuretics**				
Loop diuretics, n (%)	56 (22)	47 (22)	9 (21)	0.927 [Chi]
Furosemide (oral) dose equivalent, mg	20 (20; 40)	20 (20; 40)	20 (10; 90)	1.000 [W]
Thiazide, n (%)	73 (29)	59 (28)	14 (33)	0.460 [Chi]
Spironolactone, n (%)	14 (6)	11 (5)	3 (7)	0.607 [Chi]
**Postoperative diuretics**				
Loop diuretics, n (%)	153 (58)	125 (58)	28 (57)	0.953 [Chi]
Furosemide (oral) dose equivalent, mg	40 (20; 40)	40 (20; 40)	40 (20; 75)	0.145 [W]
Thiazide, n (%)	70 (26)	54 (25)	16 (33)	0.273 [Chi]
Spironolactone, n (%)	27 (10)	19 (9)	8 (16)	0.116 [Chi]

Pre- and postoperative need for diuretics and pre- and postoperative Furosemide (oral) dose equivalent in patients with and without postoperative de-novo atrial fibrillation. Data are presented as numbers and percentages or as median (25.; 75. percentile). [Chi] Chi-square test, [W] Wilcoxon-Mann-Whitney test.

Table S10 Pre- and postoperative diuretics in patients without severe SDB, with severe OSA and with severe CSA (SDB-groups)

	total sub-analysis cohort	no severe SDB (AHI <30/h)	severe OSA (AHI ≥30/h)	severe CSA (AHI ≥30/h)	p-value
**Preoperative diuretics**					
Loop diuretics, n (%)	56 (22)	42 (21)	3 (13)	11 (38)	0.062 [Chi]
Furosemide (oral) dose equivalent, mg	20 (20; 40)	20 (20; 50)	10 (5; 10)	20 (20; 20)	0.503 [KW]
Thiazide, n (%)	73 (29)	55 (27)	8 (35)	10 (35)	0.564 [Chi]
Spironolactone, n (%)	14 (6)	9 (4)	1 (4)	4 (14)	0.114 [Chi]
**Postoperative diuretics**					
Loop diuretics, n (%)	153 (58)	116 (55)	18 (75)	19 (59)	0.174 [Chi]
Furosemide (oral) dose equivalent, mg	40 (20; 40)	40 (20; 55)	40 (20; 40)	40 (20; 40)	0.742 [KW]
Thiazide, n (%)	70 (26)	51 (24)	7 (29)	12 (38)	0.279 [Chi]
Spironolactone, n (%)	27 (10)	16 (8)[b]	3 (13)	8 (25)[b]	**0.010 [Chi]**

Pre- and postoperative need for diuretics and pre- and postoperative Furosemide (oral) dose equivalent in patients without severe SDB, with severe OSA and with severe CSA. Data are presented as numbers and percentages or as median (25.; 75. percentile). SDB: sleep-disordered breathing, OSA: obstructive sleep apnoea, CSA: central sleep apnoea; [Chi] Chi-square test, [KW] Kruskal-Wallis test. [a] $p_{\text{severe OSA vs. no severe SDB}}$ <0.05; [b] $p_{\text{severe CSA vs. no severe SDB}}$ <0.05; [c] $p_{\text{severe OSA vs. severe CSA}}$ <0.05.

Table S11 **Multivariable logistic regression for in-hospital postoperative de-novo atrial fibrillation as dependent variable**

Independent variable	Model I (demographic model)			Model II (full model)			Model III (heart failure model)		
	**OR**	**95% CI**	**p-value**	**OR**	**95% CI**	**p-value**	**OR**	**95% CI**	**p-value**
Central apnea index-apnea index ratio	1.33	(0.48; 3.69)	0.578	1.59	(0.56; 4.55)	0.384	2.79	(0.85; 9.13)	0.090
Age ≥65 years	2.40	(1.19; 4,83)	**0.014**	2.10	(1.02; 4.35)	**0.045**	1.97	(0.89; 4.38)	0.094
Male sex	3.23	(0.94; 11.15)	0.063	3.23	(0.92; 11.36)	0.067	2.52	(0.69; 9.24)	0.163
Body mass index ≥25 kg/m^2	3.52	(1.02; 12.09)	**0.046**	3.05	(0.87; 10.65)	0.080	11.04	(1.41; 86.44)	**0.022**
Diabetes mellitus				2.35	(1.20; 4.63)	**0.013**	2.13	(0.99; 4.55)	0.051
Renal failure[†]				1.38	(0.65; 2.93)	0.401	1.44	(0.62; 3.35)	0.397
Heart failure $_{\text{GFR adjusted}}$[*]							2.13	(0.83; 5.48)	0.114

Multivariable regression analysis. Association of SDB and other preoperative predictors with postoperative in-hospital de-novo atrial fibrillation. Values are presented as OR: Odds ratio and 95% CI: confidence interval; SDB: sleep-disordered breathing. [†] glomerular filtration rate <60 ml/min/1.73 m^2; [*] n=249; NTpro-BNP $_{\text{(adjusted to GFR according to Luchner et al. Clin Chem Lab Med 2010)}}$ ≥450 pg/mL (patients < 50 years of age), ≥900 pg/mL (patients ≥50 and <75 years of age) or ≥1800 pg/mL (patients ≥75 years of age).

Table S12 Studies addressing the association between sleep-disordered breathing and postoperative de-novo atrial fibrillation after cardiac surgery

study, year	setting	type of surgery	SDB diagnosis	groups n (%)	age (years ± SD)	men (%)	BMI (kg/m²)	analysis of de-novo POAF	POAF n (%)	comments
**CONSIDER AF sub-analysis**	prospective, single center	elective CABG ± valve surgery	PG, AHI ≥30/hour	CSA 33 (12)	67.1 ± 8.0	88	28.8 + 4.7	yes	30	- severe SDB (2.23 [1.08; 4.61], p=0.030), age ≥65 years (2.17 [1.04; 4.53], p=0.038), and diabetes mellitus (2.27 [1.15; 4.48], p=0.018) were significantly associated with POAF<br>- compared to patients without POAF those with POAF had a significantly longer hospital stay (median [25.; 75. percentile]: 9 [7; 12] vs. 14 [8; 18] days, p<0.001)<br>- patients with severe CSA were affected more often by recurrent AF-episodes that were still present at 60 days post CABG than patients with severe OSA or without severe SDB (18% vs. 6% vs. 5%, p=0.039)
				OSA 24 (9)	67.8 ± 7.7	92	31.1 ± 5.0		29	
				no SDB 215 (79)	66.2 ± 8.7	82	28.4 ± 4.2		15	
Karimi et al., 2018	retrospective, databases	elective cardiac surgery	STOP-BANG questionnaire	1593 (100)	61 ± 13	67	28 ± 6	yes	37	- SDB was significantly associated with POAF (1.16 [1.09; 1.23] per point increase in the STOP-BANG-score, p<0.001)<br>- SDB was not associated with ICU length of stay (hazard ratio [97.5% CI]: 0.99 [0.96; 1.03] per point increase in the STOP-BANG-score, p=0.99)
Patel et al., 2018	retrospective, single center	CABG surgery	high risk for OSA defined by the presence of ≥3 of 5 criteria (age >65 years, hypertension, loud snoring, BMI ≥35 kg/m², Mallampati-score ≥3)	diagnosed/ treated OSA 31 (15)	67 (59; 72) *	68	NA	yes	42	- high risk for OSA was significantly associated with POAF (2.9 [1.2; 7.3], p=0.02)<br>- diagnosed/treated OSA was not associated with POAF (1.4 [0.6; 3.6], p=0.50)
				high risk OSA 39 (19)	72 (67; 76) *	74	NA		69	
				low risk OSA 139 (66)	66 (59; 76) *	74	NA		40	
Zhao et al., 2015	prospective, single center	elective CABG surgery	PAT (peripheral arterial tone)-device, AHI ≥5/hour	OSA 128 (80)	61 ± 8	86	26 ± 4	yes	25	- SDB was significantly associated with POAF (4.4 [1.1; 18.1], p=0.04)
				no OSA 32 (20)	63 ± 8	81	23 ± 3		10	
Uchoa et al., 2015	prospective, single center	elective CABG surgery	PSG, AHI ≥15/hour	OSA 37 (55)	59 ± 8	84	29 ± 4	no	22	- no difference between patient with and without OSA in the short-term follow-up<br>- POAF significantly more common in patients with than without OSA in the long-term follow up after a mean of 4.5 years
				no OSA 30 (45)	55 ± 6	63	27 ± 3		0	

Amra et al., 2014	prospective, single center	elective CABG	Berlin questionnaire	high risk OSA 25 (41)	61 ± 11	72	29 ± 4	no	8	- no difference between patients with and without high risk for OSA regarding the development of POAF
				low risk OSA 36 (59)	57 ± 10	83	26 ± 3		5	
Van Oosten et al., 2014	prospective, single center	CABG surgery	Berlin questionnaire (and PSG)	high risk OSA/ confirmed OSA 132 (48)	63 ± 10	80	31 ± 6	no	45	- high risk for OSA/confirmed OSA was significantly associated with POAF (2.18 [1.3; 3.6], p=0.003)<br>- POAF was significantly associated with increased length of hospital stay (6.5 vs. 5.3 days, p=0.007)<br>- Among patients with confirmed OSA, 22 patients (63%) were on CPAP-therapy prior to CABG surgery<br>- only patients with permanent AF were excluded from the analysis
				low risk OSA 145 (52)	66 ± 10	76	26 ± 3		30	
Mungan et al., 2013	prospective, single center	CABG surgery	Berlin questionnaire	high risk OSA 33 (45)	NA	NA	NA	yes	NA	- high prevalence for high risk OSA as diagnosed by the Berlin questionnaire in patients with POAF compared to patients without POAF (58% vs. 34%, p=0.044)
				low risk OSA 40 (55)	NA	NA	NA		NA	
Sharma et al., 2012	prospective, single center	CABG surgery	Berlin questionnaire	high risk OSA 81 (67)	60 ± 9	69	>30 in 70%	no	13	- no significant difference in patients with and without high risk for OSA regarding the development of POAF
				low risk OSA 40 (33)	59 ± 9	31	>30 in 30%		10	

Studies addressing the association between sleep-disordered breathing and postoperative de-novo atrial fibrillation in patients undergoing cardiac surgery. SDB: sleep-disordered breathing; SD: standard deviation; BMI: body mass index; POAF: postoperative atrial fibrillation; CABG: coronary artery bypass grafting; PG: polygraphy; PSG: polysomnography; AHI: apnea-hypopnea-index; CSA: central sleep apnea; OSA: obstructive sleep apnea; NA: not available; OR: Odds ratio and 95% CI: confidence interval; ICU: intensive care unit. *median (25; 75. percentile).

Table S13 Preoperative medication in patients without severe SDB, with severe OSA and with severe CSA (SDB-groups)

	total sub-analysis cohort	no severe SDB (AHI <30/h)	severe OSA (AHI ≥30/h)	severe CSA (AHI ≥30/h)	p-value
**Preoperative medication**					
Betablockers, n (%)	157 (62)	121 (60)	14 (61)	22 (76)	0.242 Chi
ACE inhibitors, n (%)	144 (56)	117 (58)	10 (43)	17 (59)	0.418 Chi
AT1 receptor antagonists, n (%)	68 (27)	52 (26)	8 (35)	8 (28)	0.637 Chi
Angiotensine receptor-neprilysin inhibitors, n (%)	1 (0)	1 (0)	0 (0)	0 (0)	0.879 Chi
Calcium channel blockers, n (%)	81 (32)	63 (31)	9 (39)	9 (31)	0.729 Chi
Anticoagulants, n	28 (11)	23 (11)	2 (9)	3 (10)	0.923 Chi

Preoperative medication in patients without severe SDB, with severe OSA and with severe CSA. Data are presented as numbers and percentages. SDB: sleep-disordered breathing, OSA: obstructive sleep apnoea, CSA: central sleep apnoea; Chi Chi-square test.

Table S14 Preoperative medication in patients with and without postoperative de-novo atrial fibrillation (POAF-groups)

	total sub-analysis cohort	no in-hospital postoperative de-novo AF	in-hospital postoperative de-novo AF	p-value
**Preoperative medication**				
Betablockers, n (%)	157 (62)	128 (60)	29 (69)	0.276 Chi
ACE inhibitors, n (%)	144 (56)	119 (56)	25 (59)	0.662 Chi
AT1 receptor antagonists, n (%)	68 (27)	57 (27)	11 (26)	0.939 Chi
Angiotensine receptor-neprilysin inhibitors, n (%)	1 (0)	0 (0)	1 (0)	**0.024** Chi
Calcium channel blockers, n (%)	81 (32)	67 (31)	14 (33)	0.811 Chi
Anticoagulants, n	28 (11)	21 (10)	7 (17)	0.197 Chi

Preoperative medication in patients with and without postoperative de-novo atrial fibrillation. Data are presented as numbers and percentages. Chi Chi-square test.

Figure S

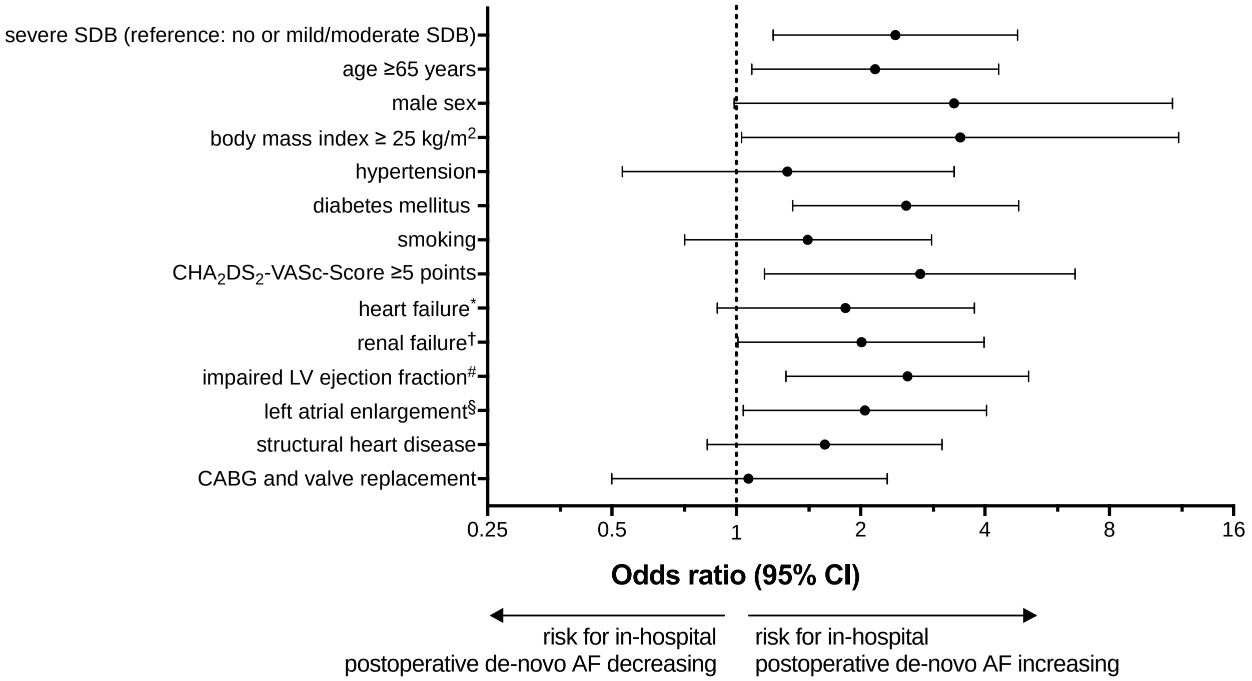

e-Figure S1 Preoperative predictors of in-hospital postoperative de-novo atrial fibrillation

Forest plot of preoperative risk factors for in-hospital postoperative de-novo atrial fibrillation. Values are presented as OR: Odds ratio and 95% CI: confidence interval. SDB: sleep-disordered breathing. * n=249; NT-proBNP (adjusted to GFR according to Luchner et al. Clin Chem Lab Med 2010) ≥450 pg/mL (patients < 50 years of age), ≥900 pg/mL (patients ≥50 and <75 years of age) or ≥1800 pg/mL (patients ≥75 years of age); † glomerular filtration rate <60 ml/min/1.73 m2; # n=250; left ventricular ejection fraction <55%; § n=228; left atrial enlargement >20cm2.
